# Early experience of dolutegravir pharmacokinetics in pregnancy: high maternal levels and significant foetal exposure with twice-daily dosing

**DOI:** 10.1097/QAD.0000000000001055

**Published:** 2016-05-03

**Authors:** Joseph M. Lewis, Eimear Railton, Andrew Riordan, Saye Khoo, Mas Chaponda

**Affiliations:** a Tropical and Infectious Diseases Unit, Royal Liverpool University Hospital; b Wellcome Trust Liverpool Glasgow Centre for Global Health Research; c Department of Paediatric Infectious Diseases, Alder Hey Children's Foundation NHS Trust; d Department of Molecular and Clinical Pharmacology, University of Liverpool, United Kingdom.

Dolutegravir is licenced for use in both adults and children over 12 years of age, although data are limited on its use in pregnant women. The manufacturer suggests that dolutegravir should be used in pregnancy only if the benefits outweigh the risks [1]. UK and US guidelines state that there are insufficient data to make recommendations on its use in pregnancy [2,3]. Nevertheless there are sporadic case reports of successful use in pregnant women [4,5] with significant placental transfer suggested by ex-vivo models [6] and a case report *in vivo*[5]. However, the effect of pregnancy on dolutegravir pharmacokinetics and optimal dosing during pregnancy is unknown. Here, we present our early experience of dolutegravir pharmacokinetics in two pregnant women, including the first published report of truncated 8-h pharmacokinetic profiles of dolutegravir in the first and third trimesters, in addition to measurement of foetal dolutegravir levels in umbilical cord blood.

Our unit has treated two pregnant women with dolutegravir, in both cases with extensive drug resistance where dolutegravir was justified to prevent onward transmission. The first was a 23-year-old woman who was referred in April 2015 for HIV management when she was 9 weeks pregnant, with a CD4 cell count of 158 cells/μl and a HIV viral load of 563 343 copies/ml. She was previously known to our services and had been well controlled on efavirenz and coformulated abacavir/lamivudine until September 2014 when she was lost to follow-up. Genotypic resistance testing showed X4 tropic virus with multiclass resistance to NRTIs and NNRTIs, but no protease or integrase resistance mutations. To aid adherence, she was commenced on a once q.d. regimen of darunavir/ritonavir (800 mg/100 mg) and dolutegravir (50 mg). Her HIV-1 viral load dropped to less than 30 copies/ml after 4 weeks. The drug was well tolerated.

Eight hour truncated pharmacokinetic profiling of dolutegravir was carried out at week 13 gestation (Fig. 1). This showed a peak dolutegravir concentration (*C*_max_) of 4006 ng/ml, in excess of the mean *C*_max_ of 3400 ng/ml in the SPRING-1 study for nonpregnant adults taking 50 mg q.d. of dolutegravir, and well above the protein-binding corrected IC_90_ of 64 ng/ml for wild-type virus [7]. Area under the curve over 0–8 h (AUC_0–8_) was 15.9 μg.h/ml.

**Fig. 1:**
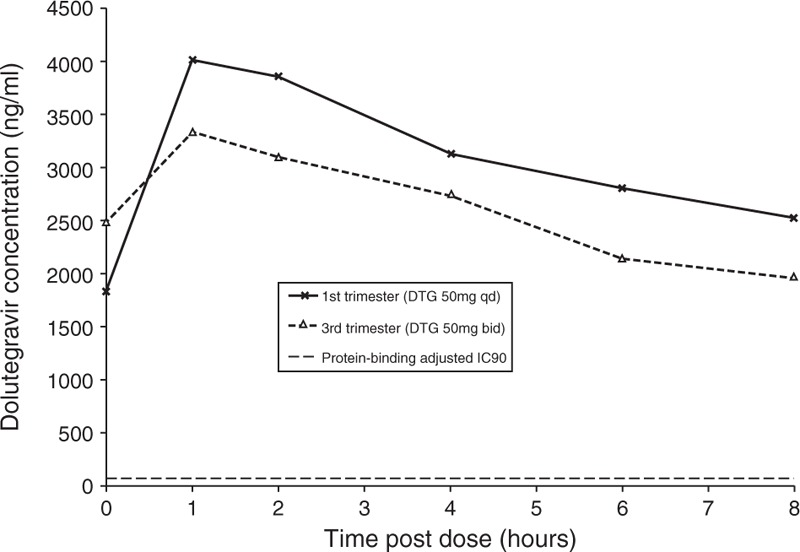
Serum dolutegravir levels pre and postdose in 1st trimester (13 weeks of gestation) and 3rd trimester (32 weeks of gestation).

DTG = dolutegravir, q.d. = once-daily, b.i.d. = twice-daily, IC90 = inhibitory concentration for 90% of viral growth. Dolutegravir concentrations were measured by liquid chromatography with tandem mass spectrometry after resolving the glucuronidated dolutegravir from parent compound.

Despite this, she experienced two rebounds in viral load to 625 and 325 copies/ml, in the context of significant medical problems (a pulmonary embolus and hydronephrosis complicated by bacteraemia). We addressed adherence concerns and her treatment was changed, at 22 weeks of gestation, to b.i.d. darunavir/ritonavir (600/100 mg) and twice daily dolutegravir (50 mg) in the anticipation that exposure to DTG would be reduced in the third trimester. Her viral load resuppressed and repeat pharmacokinetic profiling at 32 weeks of gestation (Fig. 1) showed a *C*_max_ of 3334 ng/ml, with AUC_0-8_ 13.5 μg.h/ml, a reduction of 15%.

She delivered a healthy baby by planned caesarean section at 39 weeks of gestation; dolutegravir concentrations at delivery (13 h postdose) were 1730 ng/ml in maternal blood and 2211 ng/ml in cord blood, suggesting significant in-utero exposures. On review at 8 weeks, mother and baby were well; the baby's T and B cell numbers were normal, and are thus far uninfected.

The second woman, aged 27, had poor engagement with services; she was presented in September 2015 at 31 weeks of gestation in her third pregnancy, with a CD4 cell count of 124 cells/μl and HIV-1 viral load of 235 copies/ml. She was last seen a year previously following delivery of her second child. At that time, she was virologically supressed on zidovudine, lamivudine, raltegravir, and lopinavir/ritonavir, but had subsequently defaulted from clinic attendance. In view of her late presentation and uncertain resistance profile, she was started on b.i.d. dolutegravir (50 mg) and darunavir/ritonavir (600 mg/100 mg), and once q.d. coformulated tenofovir/emtricitabine (300 mg/200 mg). This was well tolerated and 4 weeks later her HIV-1 viral load was undetectable. She delivered a healthy baby in November 2015 at 38 weeks of gestation by planned caesarean section. Dolutegravir concentration in umbilical cord blood was 1281 ng/ml, again suggesting significant in-utero exposures. On review at 6 weeks, the baby was well, and is thus far uninfected. We have reported both cases to the antiretroviral pregnancy registry (http://www.apregistry.com/Default.aspx) and follow-up of both mothers and babies is on-going.

In conclusion, we present the first detailed pharmacokinetic profile of dolutegravir in pregnancy, and confirm significant foetal exposure at a dose of 50 mg b.i.d.. The effects of this level of in-utero exposure are unknown, but we suggest caution with b.i.d. dolutegravir dosing; if this dose is used, we suggest careful follow-up to assess for toxicity in the child. Further studies of dolutegravir in pregnancy are necessary to define the dosing schedule and assess safety.

## Acknowledgements

### Conflicts of interest

There are no conflicts of interest. This work received no specific funding. J.L. is supported by the Wellcome Trust as a Wellcome Trust clinical PhD fellow (grant number 109105/Z/15/Z).
